# Dysbiosis of Gut Microbiota and Lipidomics of Content Induced by Dietary Methionine Restriction in Rice Field Eel (*Monopterus albus*)

**DOI:** 10.3389/fmicb.2022.917051

**Published:** 2022-07-07

**Authors:** Yajun Hu, Minglang Cai, Wuying Chu, Yi Hu

**Affiliations:** ^1^Hunan Engineering Technology Research Center of Featured Aquatic Resources Utilization, Hunan Agricultural University, Changsha, China; ^2^College of Animal Science and Technology, Hunan Agricultural University, Changsha, China; ^3^Department of Bioengneering and Environmental Science, Changsha University, Changsha, China

**Keywords:** gut microbiota, lipidomics, lipid metabolism, *Monopterus albus*, methionine restriction

## Abstract

An 8-week feeding trial was conducted using the rice field eel (*Monopterus albus*) with six isonitrogenous and isoenergetic experimental diets of basic feed supplemented with different levels of methionine (0, 2, 4, 6, 8, or 10 g/kg). This study built upon previous research findings that showed dietary methionine restriction (M0, 0 g/kg) inhibited hepatic fatty acid metabolism and intestinal fatty acid transportation, but both are improved by dietary supplementation with a suitable level of methionine (M8, 8 g/kg). Hence, M0 and M8 were selected to investigate how methionine regulates the gut microbiota and lipidomics of *M. albus*. Compared with M0, values for gut bacterial Sobs, Shannon, ACE, and Chao1 indices of M8 were remarkably increased (*p* < 0.05), with *Fusobacteria*, *Firmicutes*, and *Proteobacteria* the dominant phyla and *Cetobacterium*, *Plesiomonas*, and *Bacillus* the main genera in the community under the M0 vs. M8 treatments. However, compared with M0, the proportion of phyla consisting of *Fusobacteria* decreased in M8, as did the *Cetobacterium* and *Lactococcus* at the genus level; conversely, the proportions corresponding to *Firmicutes*, *Proteobacteria*, and *Chioroflexi* phyla increased in M8, as did the *Clostridium* and *Streptococcus* genera. Many edges appeared in the circus and networks, demonstrating the interspecies interactions among different operational taxonomic units (OTUs). In addition, various OTUs within the same phylum were clustered within one module. Cooperative interactions were predominant in the two networks, while competitive interactions were prevalent in their submodules. Gut microbiota mainly played roles in nutrition (lipid, amino acid, and carbohydrate) transport and metabolism under the M0 vs. M8 treatments. The PLS-DA scores indicated a significant difference in the main lipidomic components between the M0 and M8 treatment groups. Namely, the TG(26:0/16:0/17:0), TG(28:0/16:0/16:0), TG(26:0/16:0/16:0), and TG(30:0/16:0/16:0)—among others—comprising the gut content were reduced under the M8 treatment (*p* < 0.001). The genus *Clostridium* was positively correlated with TG(18:1/18:1/22:5), TG(16:0/17:0/18:1), TG(18:0/18:1/20:3), and other compounds, yet negatively correlated with TG(18:0/17:0/20:0), TG(16:0/17:0/24:0), and TG(16:0/16:0/24:0), among others as well. According to the lipidomics analysis, the predicted KEGG pathways mainly included lipid and glycan biosynthesis and metabolism, and digestive, sensory, and immune systems. In conclusion, methionine restriction disturbed the microbial community balance and induced microbial dysfunctions, whereas methionine supplementation improved the homeostasis of gut microbiota and lipid metabolism of the rice eel.

## Introduction

The gut cavity is home to a diverse and abundant microbiome, which has a pivotal role in maintaining the host’s physiological homeostasis (Xu et al., 2020). Gut microbiota have co-evolved with their hosts and have metabolic characteristics enabling them to contribute to a host’s metabolism (Gaulke et al., 2018). The gut microbial composition is known to be influenced by environmental, dietary, and physiological factors associated with the host, these varying among types of hosts, which mainly alters the species and abundance of microbiota and microbial community composition (Marchix et al., 2018; Peng et al., 2019b). Importantly, the changed commensal microbiota could further affect the host’s immunity, metabolism, and behavior (Kamada et al., 2013; Ridaura et al., 2013; Henriques et al., 2020). The stability of microbial ecological network could be affected by the host’s microbial species and their relative abundances; moreover, within the microbial community and its ecological network, interspecific interactions can have consequences for the dynamic balance, systemic functioning, and metabolism and health of the host (Yang et al., 2019a). Further, the gut microbiome also been considered to function as a metabolic organ, one involved in carbohydrate, energy, lipid, and amino acid metabolism activities that play positive or negative roles in the host (Wang et al., 2020).

Gut microbiota can affect their host’s lipid metabolism *via* multiple direct and indirect biological mechanisms (Ghazalpour et al., 2016); hence, some have argued the gut microbial community constitutes an endocrine organ (Clarke et al., 2014). Semova et al. (2012) used fluorescent markers to image zebrafish (*Barchydanio rerio* var.), finding that its gut microbiota could stimulate the uptake of fatty acids and formation of lipid droplets by the intestinal epithelium and liver, with the sclerenchyma enhancing absorption capacity of fatty acids by host’s intestinal cells, thereby promoting an increase in the number of lipid droplets. Meanwhile, the size of these lipid droplets increased as bacterial abundance increased, which the authors demonstrated was primarily regulated by golden rod bacteria (*Chrysobacterium hispanicum*) and *Pseudomonas* sp. (*P. adaceae*). Later, Almeida et al. (2019) reported that gut bacteria could ferment indigestible carbohydrates, and then digest the indigestible fiber of gut contents into short-length chain fatty acids. Additionally, gut microbiota could promote the absorption of fatty acids by activating the absorption capacity of intestinal epithelial cells.

The rice field eel (*M. albus*) is subtropical freshwater benthic fish with considerable economic value, which is why it is widely raised in central and south China (Hu et al., 2020b). In their natural habitat, *M. albus* can prey on earthworms, frog eggs, and insects (Duan et al., 2018). Because it has a straight tubular gastrointestinal system, whose stomach and intestine are easily distinguished, this organism is an ideal experimental object to explore the succession of gut microbiota. Our previous study of *M. albus* indicated that a high-fat feed is capable of affecting the succession of its gut microbiota community, by disturbing the balance of gut microbiota and reducing the average connectivity and number of connectors, as well as competitive interactions, within the ecological network (Peng et al., 2019b). In other work, we also found that oxidized fish oil feed strongly affected the species of gut microbiota, inducing microbial dysbiosis, which led to microbial dysfunction in *M. albus*; but taurine supplementation of oxidized fish oil feed improved the community stability of gut microbiota, ameliorating the negative effects induced by oxidized fish oil diet, and restoring the relevant functioning of gut microbiota (Peng et al., 2019a).

We had reported that *M. albus* requires an optimal protein/lipid ratio diet (Ma et al., 2014). In addition, we found that when fish meal is replaced by soybean meal (Hu et al., 2018) and soy protein concentrate (Zhang et al., 2019), this inhibited the growth performance of *M. albus*. Yet a diet deficient in methionine also inhibits the growth performance of *M. albus* and reduces hepatic lipid bodies’ deposition, resulting in less hepatic fatty acid synthesis (Hu et al., 2021a). Furthermore, insufficient methionine intake also damaged the gastric and intestinal structure, reduced the function of intestinal barrier, and inhibited the ability of intestinal lipid and fatty acid transportation of *M. albus* (Hu et al., 2022a). In mice on a high-fat diet, however, suitable methionine restriction improved the gut’s function by regulating the microbiota there and its metabolite profiles (Yang et al., 2019b). Yet how gut microbial community changes adaptively and regulates host’s metabolism in response to methionine restriction remains unknown, especially in aquatic animals.

Here, following our previous study (Hu et al., 2021b), soy protein concentrate was used in basic fish meal to make a severely methionine-deficient experimental feed. The objectives were (1) to explore gut microbiome dynamics in terms of microbial composition, succession, interactions, and network topological roles, and then predict the gut microbial functioning in *M. albus*, and (2) to use lipidomics to reveal how gut microbiota affect the host’s lipid metabolism and absorption. This study demonstrates the alteration of the microbiome and lipid-related products in response to methionine restriction, and thus may provide a new perspective on the theory of lipid metabolism.

## Materials and Methods

### Experimental Diets

A basic diet (110 g/kg fish meal; 400 g/kg soy protein concentrate), as prepared in previous studies (Zhang et al., 2019; Hu et al., 2021b), was supplemented with different levels of methionine (0, 2, 4, 6, 8, or 10 g/kg). Table 1 shows the composition and nutrition of these methionine diets.

**Table 1:** Composition of the six diets and their nutritive concentrations (g/kg).

Ingredients	M0	M2	M4	M6	M8	M10
Fish meal	110	110	110	110	110	110
Soy protein concentrate	400	400	400	400	400	400
Fish oil	40	40	40	40	40	40
^1^DL-Methionine	0	2	4	6	8	10
Lysine	3.6	3.6	3.6	3.6	3.6	3.6
Glycine	16	14	12	10	8	6
Glutamate	4	4	4	4	4	4
^2^Attractant	1	1	1	1	1	1
Wheat meal	138.4	138.4	138.4	138.4	138.4	138.4
α-starch	200	200	200	200	200	200
Brewer yeast	50	50	50	50	50	50
Choline chloride	5	5	5	5	5	5
Ca(H_2_PO_4_)_2_	20	20	20	20	20	20
^3^Vitamin and mineral premix	12	12	12	12	12	12
Total	1,000	1,000	1,000	1,000	1,000	1,000
**Proximate analysis**						
Dry matter (g/kg)	922.66	925.27	928.12	928.43	923.63	924.78
Crude protein (g/kg)	445.92	443.41	458.73	447.40	451.84	450.77
Crude lipid (g/kg)	67.86	67.11	68.69	67.70	67.92	68.07
Crude ash (g/kg)	102.60	101.90	100.60	102.60	101.90	100.60
Gross energy (kJ/g)	19.10	18.86	18.74	19.17	19.25	19.10

1 DL-Methionine (BR, 99%) was purchased from the Shanghai Yuanye Biotechnology Co., Ltd. (Shanghai, China).

2 Attractant: 40% betaine; 20% DMPT; 20% threonine; 10% glycine; 10% inosine-5′-diphosphate trisodium salt.

3 This vitamin and mineral premix was provided by the MGOTer Bio-Tech Co. Ltd. (Qingdao, Shandong, China). Its composition was as follows (mg/kg diet): KCl, 200 mg; KI (1%), 60 mg; CoCl_2_·6H_2_O (1%), 50 mg; CuSO_4_·5H_2_O, 30 mg; FeSO_4_·H_2_O, 400 mg; ZnSO_4_·H_2_O, 400 mg; MnSO_4_·H_2_O, 150 mg; Na_2_SeO_3_·5H_2_O (1%), 65 mg; MgSO_4_·H_2_O, 2,000 mg; Zeolite powder, 3645.85 mg; VB_1_, 12 mg; riboflavin, 12 mg; VB_6_, 8 mg; VB_12_, 0.05 mg; VK_3_, 8 mg; inositol, 100 mg; pantothenic acid, 40 mg; niacin acid, 50 mg; folic acid, 5 mg; biotin, 0.8 mg; VA, 25 mg; VCP_1_, 5 mg; VE, 50 mg; VC, 100 mg; ethoxyquin, 150 mg; wheat meal, 2434.15 mg.

The proximate analysis (moisture, crude lipid, crude protein, ash, and gross energy) of the experimental feed treatments was determined based on our previous paper (Hu et al., 2021c). Amino acids (Table 2) were analyzed by an automatic amino acid analyzer (Agilent-1,100, Agilent Technologies Co., Ltd., Santa Clara, CA, United States), by referring to the methodology of Wijerath Wiriduge et al. (2020), while fatty acids (Table 3) were analyzed by GC–MS (Agilent 7890B-5977A, Agilent Technologies Co., Ltd., Santa Clara, CA, United States) according to Jin et al. (2017).

**Table 2:** The amino acid content (g/kg) of the six experimental diets.

Amino acids	M0	M2	M4	M6	M8	M10
His^*^	9.787	9.629	9.926	9.727	9.996	9.768
Ser	18.942	18.519	19.070	18.690	18.904	18.570
Arg^*^	23.417	23.854	23.425	23.199	23.535	23.118
Gly	32.731	30.514	28.362	26.275	24.132	22.012
Asp	42.245	42.158	42.106	42.711	42.535	42.631
Glu	75.484	75.673	75.215	75.742	75.918	75.681
Thr^*^	15.514	15.230	15.556	15.925	15.412	15.881
Ala	19.718	19.301	19.759	19.447	19.697	19.424
Pro	20.227	19.697	20.153	20.330	20.575	20.228
Cys	1.084	1.029	1.088	1.091	1.084	1.094
Lys^*^	36.887	36.186	36.894	36.382	36.818	36.248
Tyr	9.802	9.759	9.852	9.040	9.397	9.634
Met^*^	1.860	3.781	5.920	7.739	9.609	11.525
Val^*^	18.640	18.211	18.637	18.379	18.590	18.323
Ile^*^	17.478	17.136	17.618	17.638	17.890	17.465
Leu^*^	29.125	29.612	29.267	29.666	29.493	29.420
Phe^*^	18.457	18.104	18.558	18.220	18.565	18.100
Trp	/	/	/	/	/	/

* An essential amino acid; Trp not detected.

**Table 3:** The fatty acid content (mg/100 g) of the six experimental diets.

Fatty acid	M0	M2	M4	M6	M8	M10
C4:0	13.21	13.72	14.49	13.53	13.15	14.16
C8:0	5.07	5.08	4.91	5.05	5.04	5.00
C12:0	3.13	3.64	4.34	3.35	3.35	4.37
C13:0	11.13	10.39	9.71	11.29	10.32	10.14
C14:0	181.39	183.69	182.55	182.37	183.62	182.57
C14:1	2.19	2.62	2.81	2.88	2.70	2.83
C15:0	19.90	20.22	20.52	19.93	20.21	20.51
C16:0	609.04	608.96	606.58	609.36	608.55	606.84
C16:1	6.46	7.59	6.88	6.56	7.58	6.88
C17:0	12.58	13.74	13.65	12.80	13.42	13.52
C17:1	6.27	6.91	7.33	6.73	6.97	7.38
C18:0	120.68	121.92	121.78	121.68	121.97	121.80
18:1-T	16.16	16.09	17.89	16.10	16.02	17.86
C18:1 N9C	415.27	410.17	418.66	413.30	410.15	418.53
18:2-T	2.74	3.35	2.45	2.73	3.34	2.46
C18:2N6C	17.35	16.63	18.71	18.34	16.86	18.12
C20:0	11.13	10.45	10.49	10.30	10.40	10.42
C20:1	25.44	27.37	27.27	23.43	27.34	27.22
C18:3 N3	235.71	235.00	236.16	235.11	236.65	235.11
C20:2	10.35	10.88	10.31	10.36	10.85	10.34
C22:0	5.84	5.85	5.95	5.39	5.88	5.91
C22:1 N9	197.83	197.62	194.40	197.33	197.65	196.49
C20:3 N3	32.37	31.17	34.19	32.74	33.13	34.16
C20:4 N6	25.20	25.82	25.45	25.57	25.18	25.40
C24:0	248.36	249.92	237.64	248.40	249.18	237.43
C20:5 N3	101.77	100.98	101.88	101.17	101.90	101.89
C24:1	21.19	21.36	22.29	21.39	21.32	23.23
C22:6 N3	575.88	571.14	571.93	575.90	571.16	570.93

### Feeding Trial and Management

Individuals of *M. albus* were purchased from Changde, China. Similar-sized *M. albus* (25.08 ± 0.31 g) were reared in 18 float cages (2.0 m × 1.5 m × 1.5 m), three per dietary treatment (triplicates), with 60 fish per cage. More details about the experiment can be found in our recent paper (Hu et al., 2022b).

### Ethics Statement

Our study was approved by the Committee of Laboratory Animal Management and Animal Welfare of Hunan Agricultural University (Changsha, China) {No. 094}. All experimental fish were anesthetized with eugenol (1:12,000; Shanghai Reagent Corporation, Shanghai, China) to reduce their suffering.

### Sample Collection and Analyses

Our previous research showed that a methionine-deficient diet (i.e., M0, 0 g/kg) inhibited hepatic fatty acid metabolism (Hu et al., 2021a) and intestinal fatty acid transportation (Hu et al., 2022a), but a suitable level of supplemented methionine (M8, 8 g/kg) enhanced hepatic lipid metabolism and intestinal fatty acid transportation. Hence, M0 (0 g/kg) and M8 (8 g/kg) were focused upon to elucidate the mechanisms by which methionine intake regulates the gut microbiota and lipidomics of *M. albus*. After the 8-week feeding trial, from each cage the gut contents of five fish were sampled, and these five samples under each experimental group were used for the sequencing analysis.

### Gut Microbiota Analysis by 16S rRNA Sequencing

DNA was obtained from the gut content by using the PowerFecal^™^ DNA Isolation Kit (MoBio Laboratories, Inc.). High-throughput sequencing was performed on the Illumina MiSeq platform, in which all sequences were classified into operational taxonomic units (OTUs) at a minimum 97% similarity by the QIIME (Quantitative Insights Into Microbial Ecology) software pipeline, after first removing any low-quality scores (Q score, 20) with the FASTX-Toolkit (Hannon Lab, United States). For full details about the bioinformatics analysis and molecular ecological network construction, please refer to our previous study (Peng et al., 2019b).

### Untargeted Lipidomics by the LC–MS Platform

The samples were analyzed by liquid chromatography, whereby a single component entered the ion source of the high-vacuum mass spectrometer for ionization. The mass spectrum is obtained by separation according to the mass-to-charge ratio (*m/z*). Finally, the qualitative and quantitative results of each sample were obtained *via* its mass spectrum data analysis. LC–MS platform used was the UHPLC-Q Exactive system (Thermo Fisher Scientific, Waltham, Massachusetts, United States; Huang et al., 2022).

### Statistical Analysis

Our data were analyzed on an online cloud platform of Majorbio (ShangHai Majorbio Bio-pharm technology Co., Ltd.).^1^ For the M0 and M8 data, their means for a given response variable were compared by an independent *t*-test in SPSS 22 software. Results are presented as the mean ± SEM (standard error of the mean), with differences considered significant at *p* < 0.05.

## Results

### Gut Bacterial Diversity Indices

Both the Coverage and Simpson were similar between the M0 (0g/kg) and M8 (0g/kg) treatment groups. Compared with M0, gut bacterial Sobs, Shannon, ACE, and Chao1 for M8 were significantly increased (*p* < 0.01, *p* < 0.01, *p* < 0.05, *p* < 0.05, respectively; Table 4).

**Table 4:** Effects of methionine restriction on gut bacterial diversity indices after feeding for 8 weeks (*n* = 3).

Indices	M0	M8	*P* value
Coverage	1 ± 0	1 ± 0	0.205
Sobs	81.5 ± 5.5	330 ± 2	0.006
Shannon	1.69 ± 0.04	2.83 ± 0.04	0.002
Simpson	0.8 ± 0.03	0.79 ± 0.06	0.823
ACE	196.96 ± 4.26	367.58 ± 10.35	0.019
Chao1	131.58 ± 1.42	359.15 ± 9.79	0.024

Values are the means ± SEM, not significantly different if *p* > 0.05.

### Gut Bacterial OTUs and Their Relative Abundances

**Figure 1:**
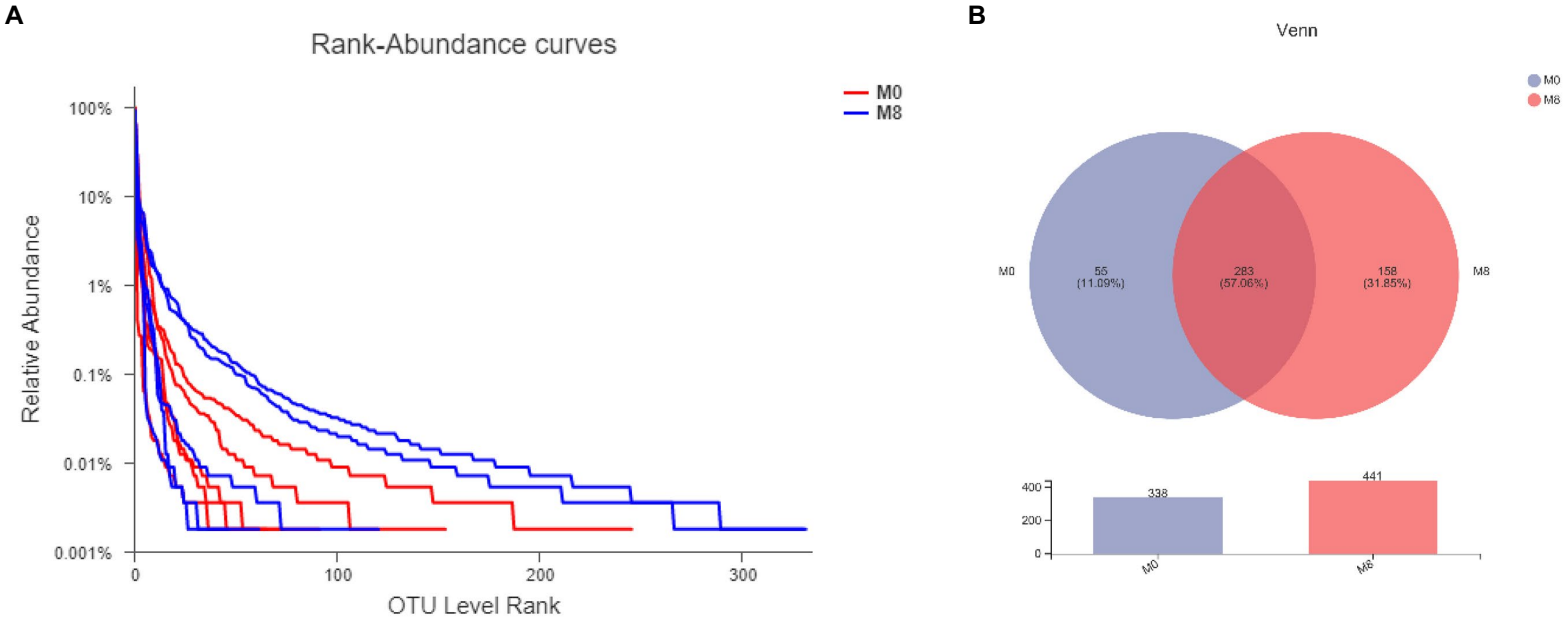
Effects of methionine restriction on gut bacterial numbers of shared OTUs based on core analysis **(A)** and gut bacterial relative abundances **(B)** after feeding for 8 weeks (*n* = 3).

When compared with M0, the trend is of species diversity being higher in the M8 samples (Figure 1A). There are 283 species common to both M0 and M8 groups, and 338 species overall in M0, of which 55 are unique to M0, and 441 species overall in M8, of which 158 are unique to M8 (Figure 1B).

### Gut Bacterial Composition at the Phylum and Genus Levels

**Figure 2:**
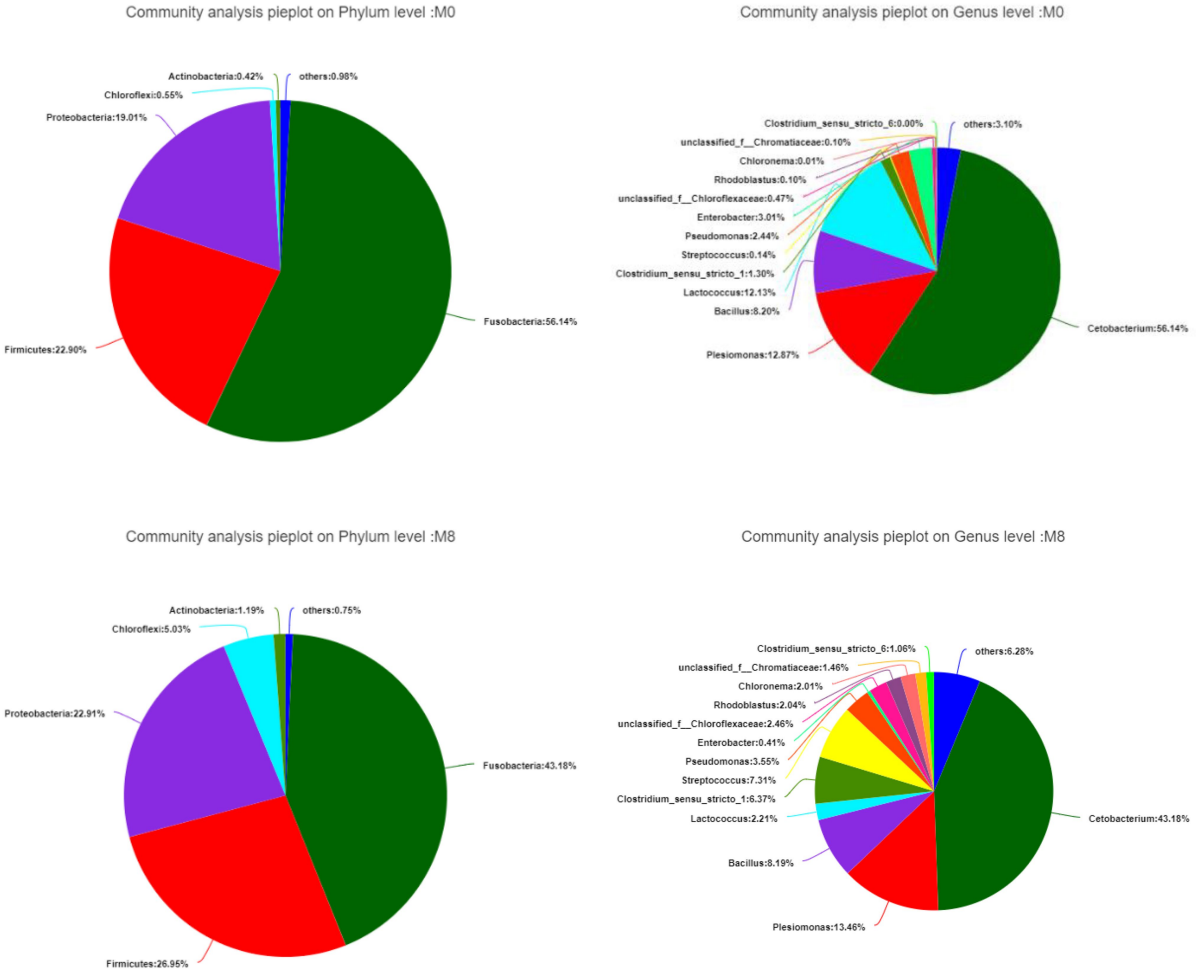
Effects of methionine dietary restriction on gut bacterial composition at the phylum (left) and genus (right) levels after feeding for 8 weeks (*n* = 3).

*Fusobacteria* was the main phylum member of the community while *Cetobacterium* was the dominant genus. When compared with the M0 group, the proportions belonging to the *Fusobacteria* phylum and *Cetobacterium* and *Lactococcus* genera all decreased in M8; by contrast, *Firmicutes*, *Proteobacteria,* and *Chioroflexi* phyla were increased in M8, as were the genera *Clostridium* and *Streptococcus* (Figure 2).

### Ecological Network Analysis

**Figure 3:**
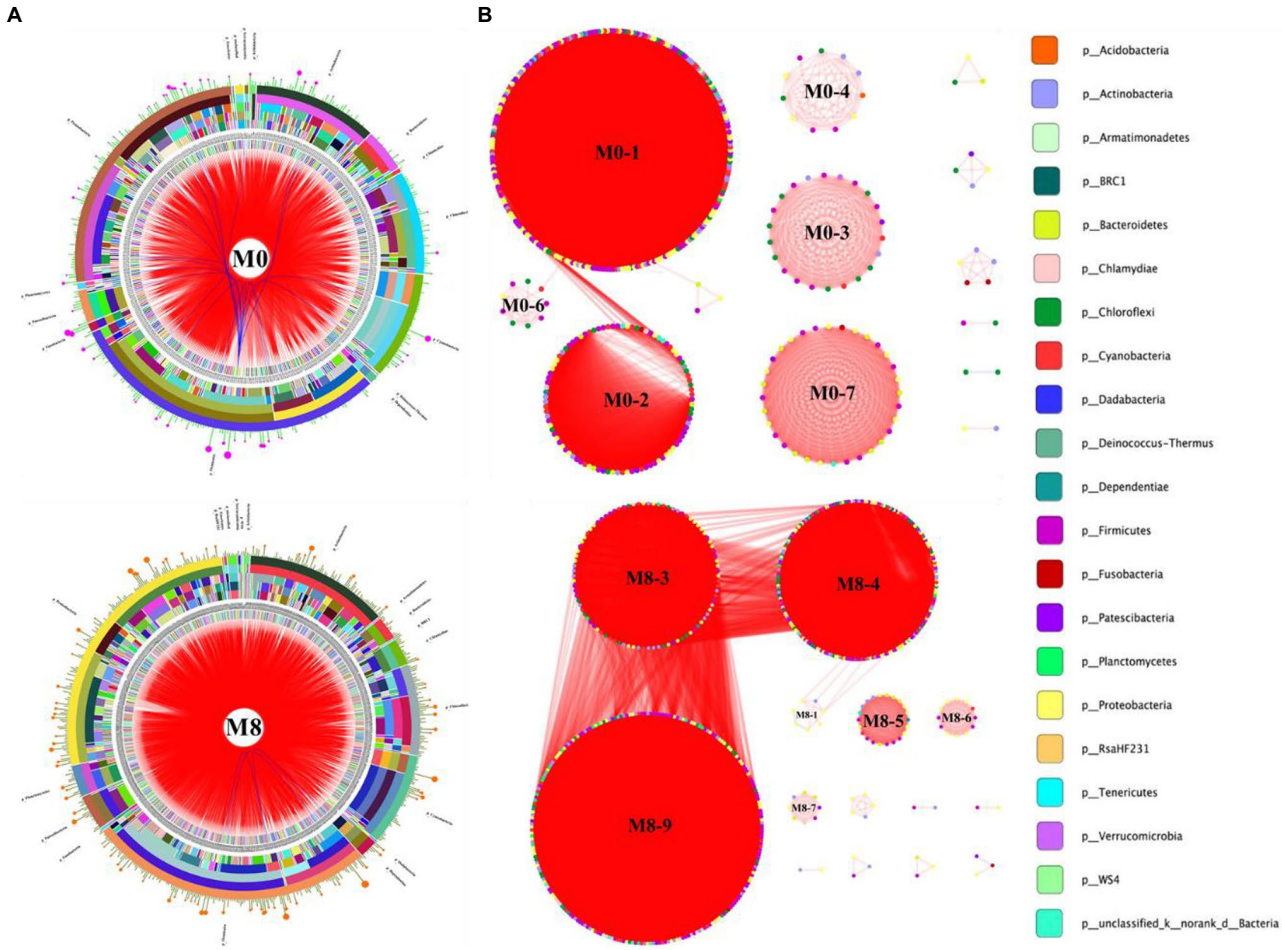
Circos plots **(A)** showing the assignment of OTUs at different taxonomic levels of classification. Ecological network **(B)** showing the submodules and interspecific interactions in the gut bacterial community of *M. albus*. The data were analyzed using the R Programming Language. The taxonomic levels were phylum, class, order, family, and genus, moving from the outside to the inside of the circle, respectively. Bands differing in color show different genera, and the bar width indicates the abundance of each taxon in the circos plot. The modular organization was constructed by implementing the modularity optimization method. Each node in the network graph corresponds to a single OTU. Colors of the nodes indicate different major phyla. The edges inside the circle and ecological network represent the interactions between species (pink edge, positive interaction; blue and red edges, negative interactions).

As Figure 3 shows, the dominant phyla in the M0 group were *Firmicutes*, *Proteobacteria*, *Actinobacteria*, *Chloroflexi,* and *Cyanobacteria*; those dominant in M8 were *Firmicutes*, *Proteobacteria*, *Actinobacteria*, *Chloroflexi*, and *Cyanobacteria*. Hence, dominant taxa were similar between the networks for M0 and M8. Many edges in the circus and networks revealed interspecies interactions among the different OTUs. In addition, six submodules were discernible in the M0 network, whose three largest submodules were M0-1, M0-2, and M0-7. Likewise, six submodules were detected in the M8 network, the three largest submodules being M8-9, M8-4, and M8-3. These OTUs from the same phylum were clustered in a single module; cooperative interactions were predominant in both networks, whereas competitive interactions characterized their submodules, such as M0-7, M0-3, M0-4, and M0-6 of the M0 network, and M8-5, M8-6, and M8-7 of the M8 network (Figure 3).

### Gut Bacterial Function: Wilcoxon Rank-Sum Tests

**Figure 4:**
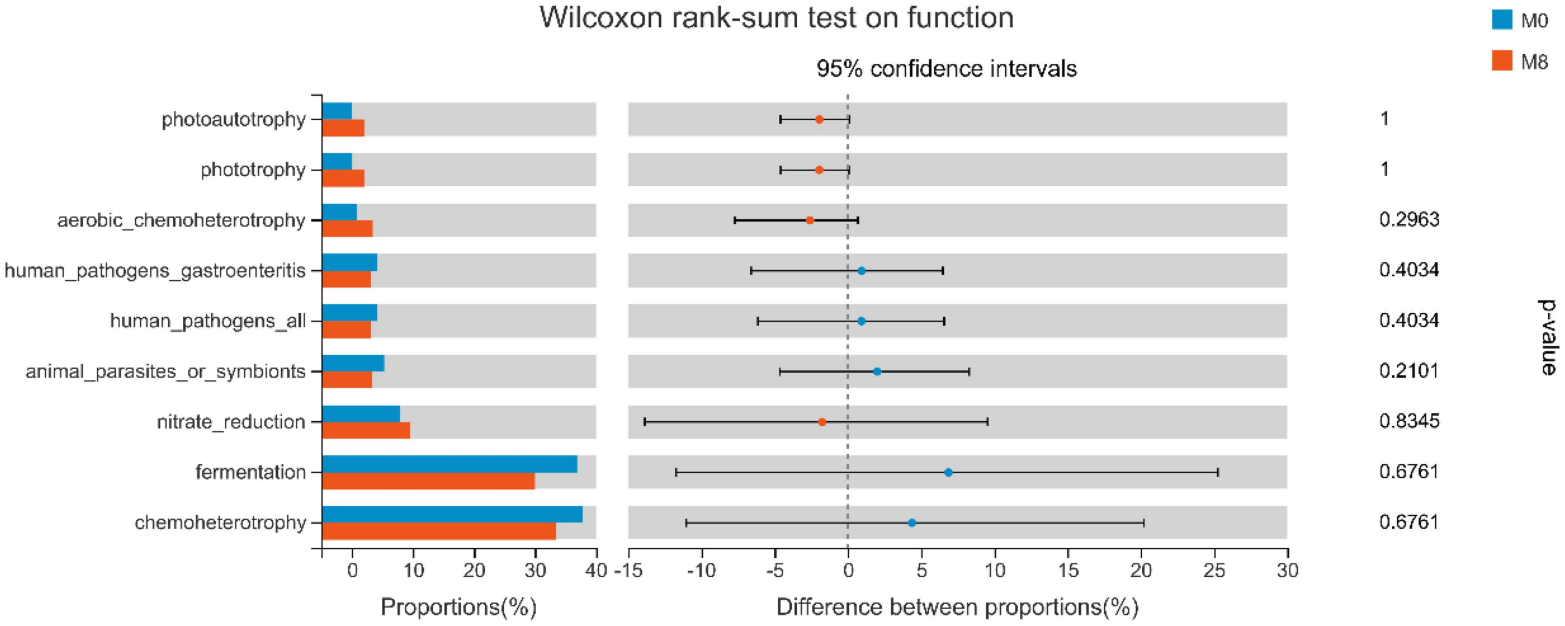
Wilcoxon rank-sum tests for the effects of methionine restriction on gut bacterial functioning after feeding for 8 weeks (*n* = 3). Values are considered not significantly different if *p* > 0.05.

In Figure 4, human pathogens gastroenteritis, human pathogens, animal parasites or symbionts, nitrate reduction, fermentation and chemoheterotrophy were lower in M8 than M0, and vice versa for photoautotrophy, phototrophy, aerobic-chemoheterotrophy, and nitrate reduction.

### Gut Bacterial COG Functional Classification

**Figure 5:**
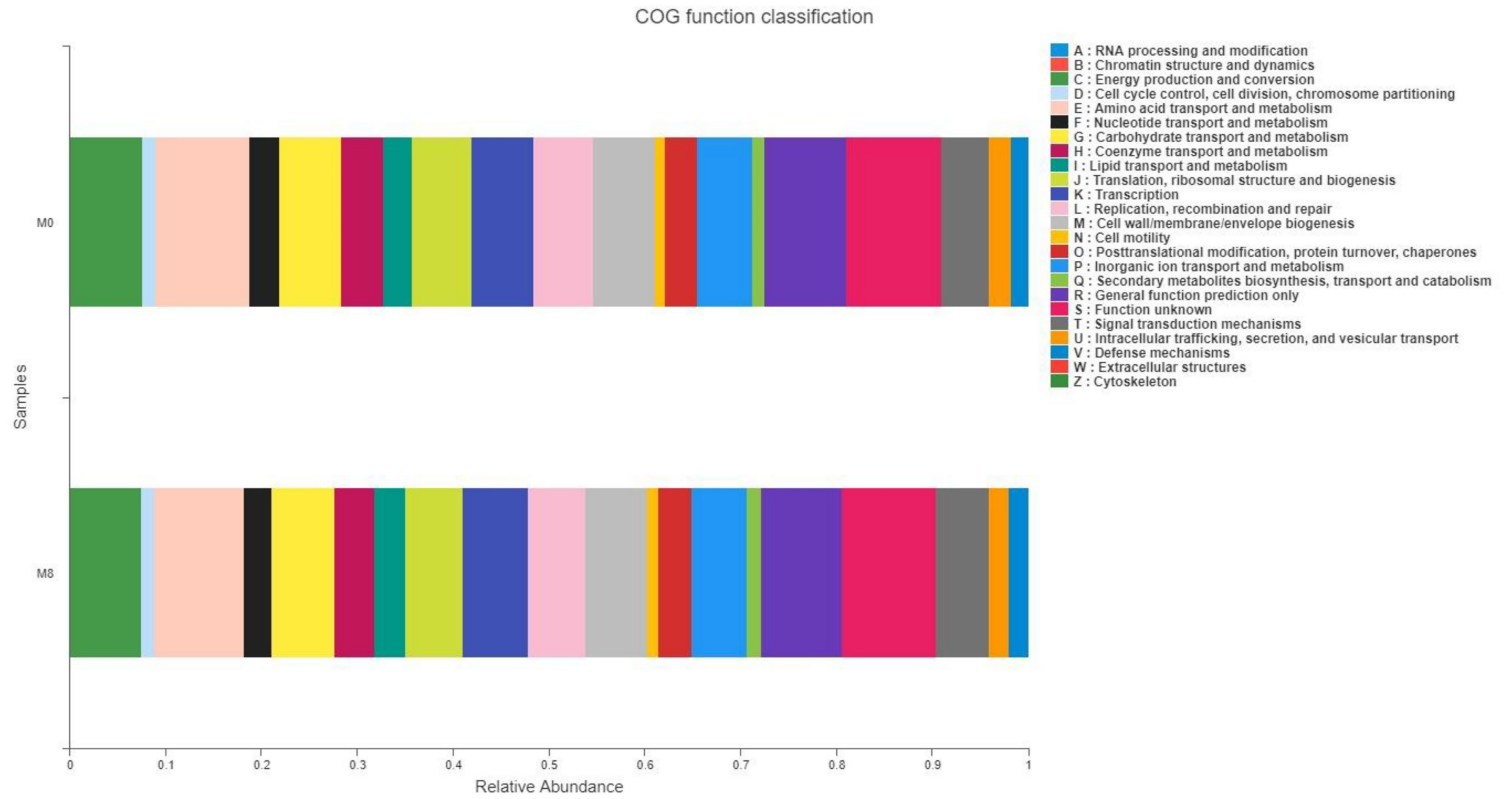
Effects of methionine restriction on COG functional classification of gut bacteria after feeding for 8 weeks (*n* = 3).

As seen in Figure 5, we observed that gut microbiota mainly played roles in energy production and conversion, Figure 5 and nutrition (lipid, amino acid, and carbohydrate) transport and metabolism processes.

### Venn Diagram of Lipidomics

**Figure 6:**
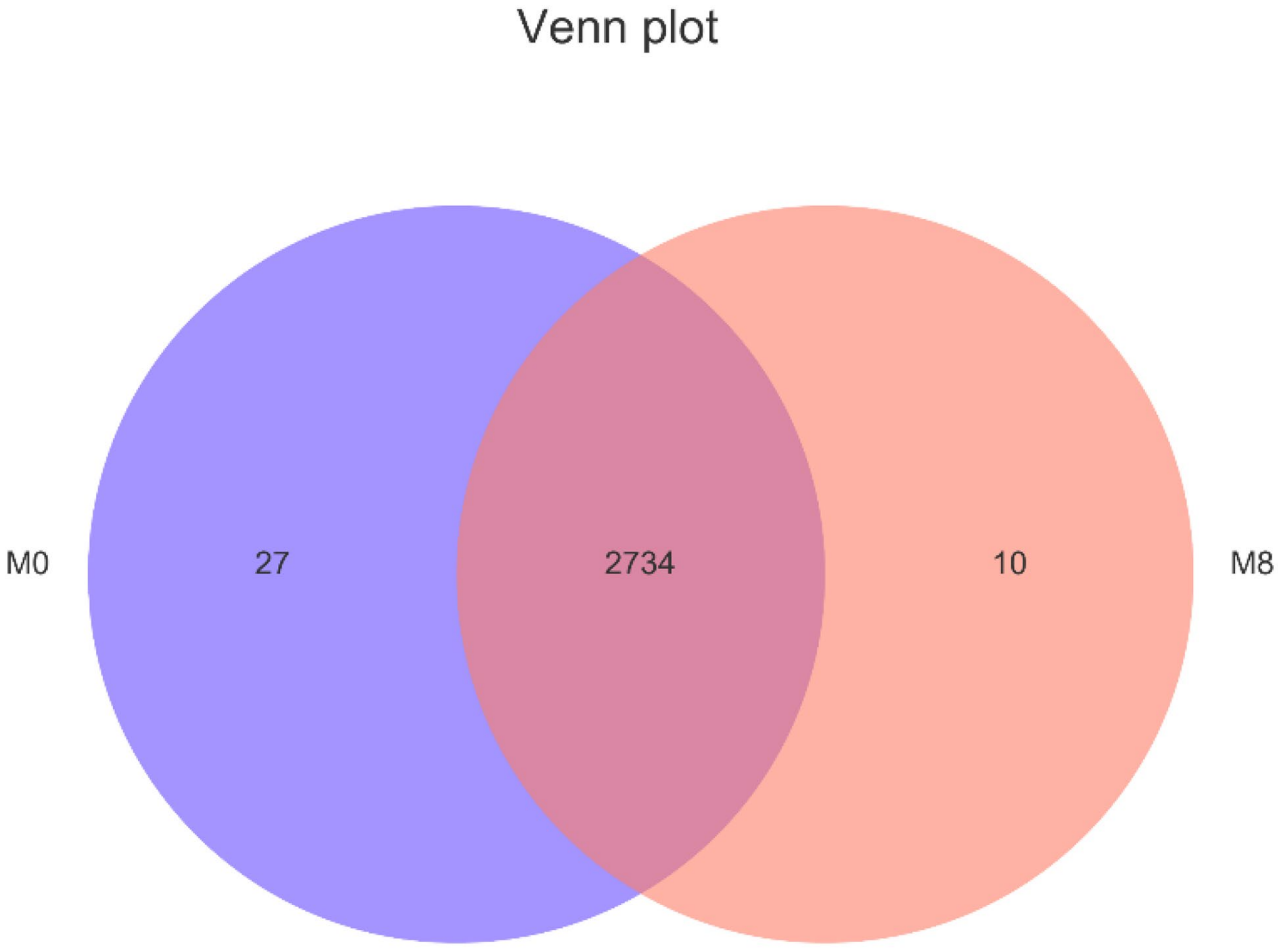
Effects of methionine restriction on different on lipidomics after feeding for 8 weeks (*n* = 3).

There were 2,734 lipids common to M0 and M8 groups, with 2,761 lipids overall found in M0, of 27 were unique to it; likewise, 2,744 lipids were found in M8, with 10 of these unique to it (Figure 6).

### PCA and PLS-DA of Lipidomics

**Figure 7:**
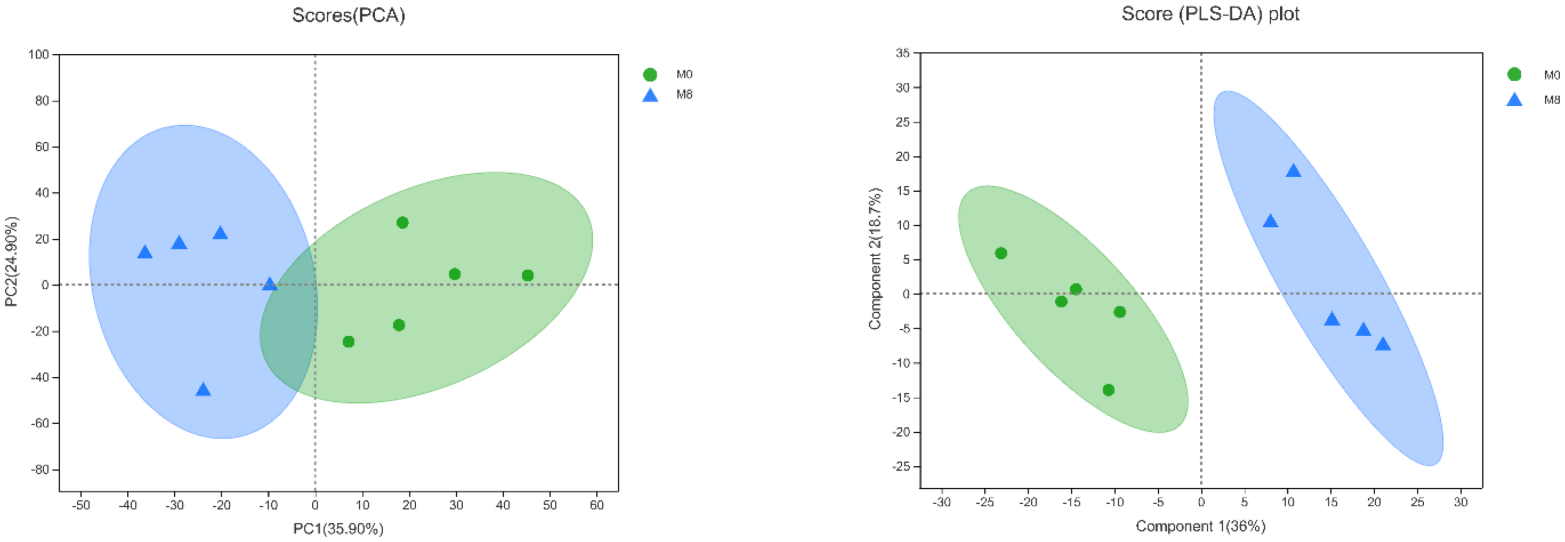
Effects of methionine restriction according to a principal component analysis (PCA) and partial least squares-discriminant analysis (PLS-DA) of the lipidomics data after feeding for 8 weeks (*n* = 3).

The PCA scores showed that the degree of dispersion for the lipidomics was very high between M0 and M8. Further, the PLS-DA scores indicated significant differences in the main components of lipids between the M0 and M8 treatment groups (Figure 7).

### Expression Profiles and VIP of Metabolites

**Figure 8:**
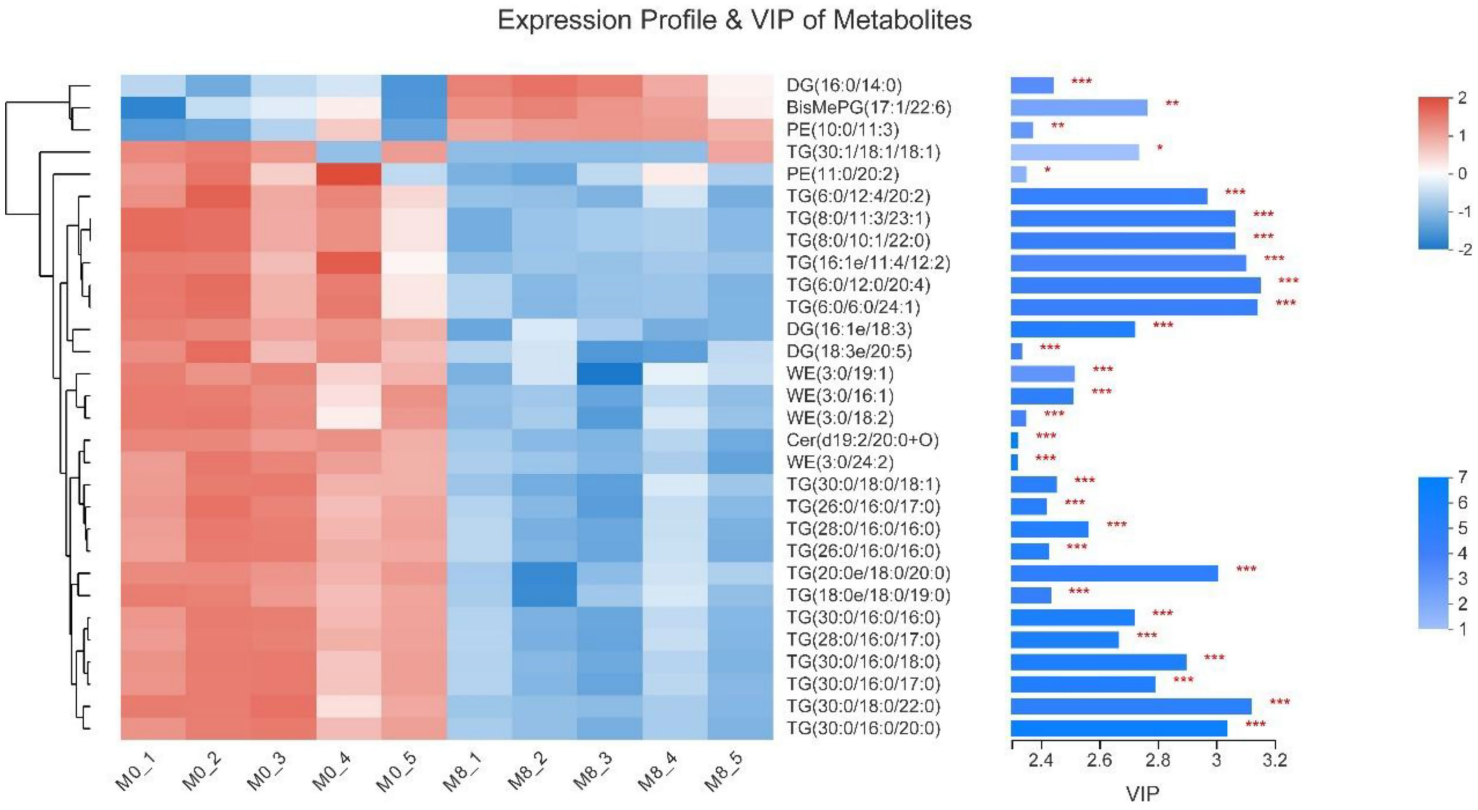
Effects of methionine restriction on the expression profile and VIP of metabolites obtained *via* lipidomics after feeding for 8 weeks (*n* = 3); ^*^p < 0.05, ^**^p < 0.01, and ^***^p < 0.001.

Compared with M0, DG(16:0/14:0), BisMePG(17:1/22:6) and PE(10:0/11:3) of gut content in M8 were remarkably increased (*p* < 0.001, *p* < 0.01, and *p* < 0.01, respectively); while TG(30:1/18:1/18:1) and PE(11:0/20:2) of gut content in M8 (Met5) were remarkably decreased (*p* < 0.05), TG(6:0/12:4/ 20:2), TG(8:0/11:3/23:1), TG(8:0/10:1/22:0), TG(16:1e/11:4/ 12:2), TG(6:0/12:0/20:4), TG(6:0/6:0/24:1), DG(16:1e/18:3), DG(18:3e/20:5), WE(3:0/19:1), WE(3:0/16:1), WE(3:0/18:2), Cer(d19:2/20:0 + O), WE(3:0/24:2), TG(30:0/18:0/18:1), TG(26:0/16:0/17:0), TG(28:0/16:0/16:0), TG(26:0/16:0/16:0), TG(20:0e/18:0/20:0), TG(18:0e/18:0/19:0), TG(30:0/16:0/16:0), TG(28:0/16:0/17:0), TG(30:0/16:0/18:0), TG(30:0/16:0/17:0), TG(30:0/18:0/22:0) and TG(30:0/16:0/20:0) of gut content in M8 (Met5) were remarkably decreased (*p* < 0.001; Figure 8).

### Correlation Analysis of Microbiota and Lipidomics Data

**Figure 9:**
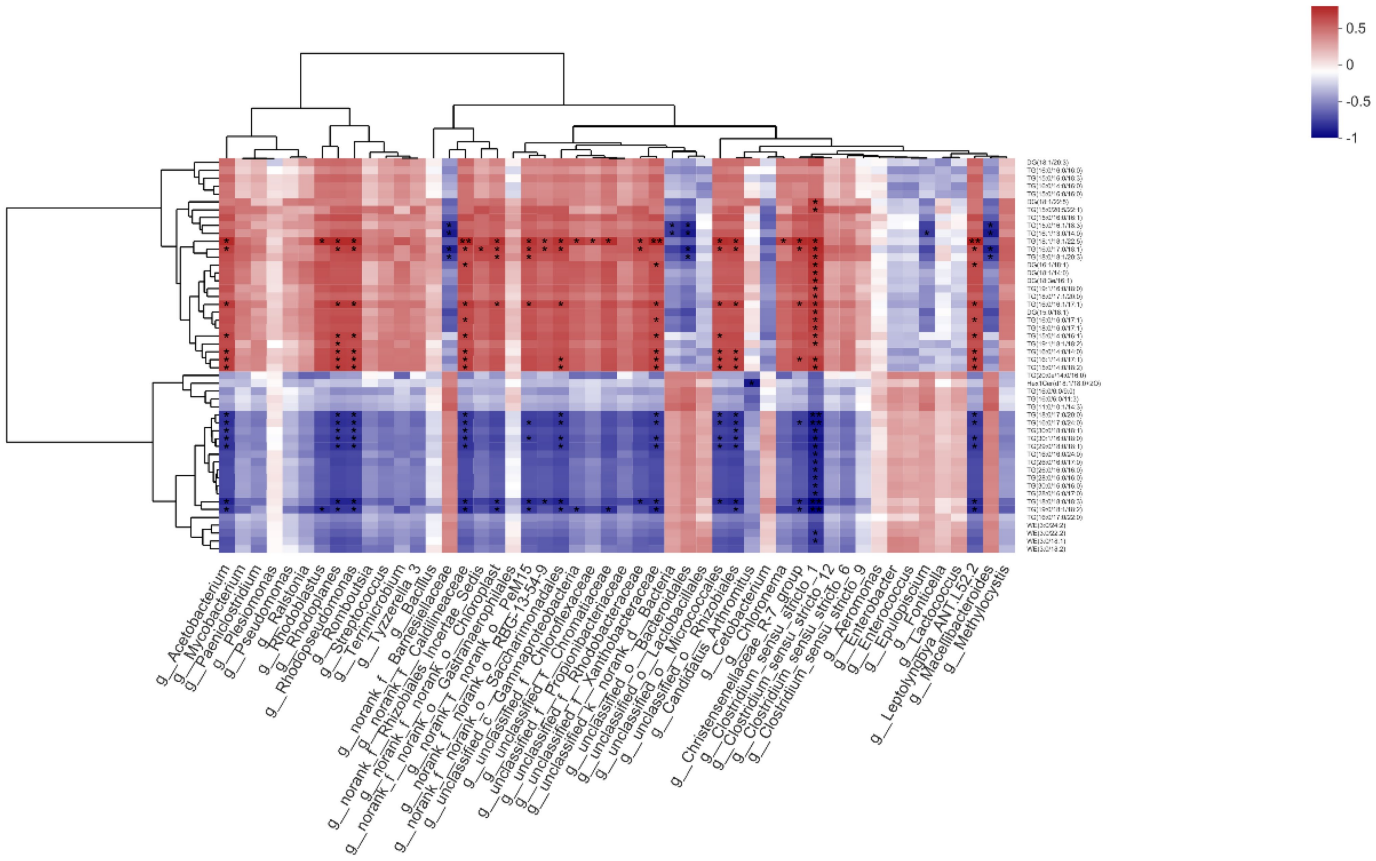
Effects of methionine restriction on the correlations between specific microbiota and lipids after feeding for 8 weeks (*n* = 3); ^*^p < 0.05 and ^**^p < 0.01.

The g__*Acetobacterium* had significant positive correlations with TG(18:1/18:1/22:5), TG(16:0/17:0/18:1), TG(16:0/16:1/17:1), TG(15:0/14:0/16:1), TG(16:0/14:0/14:0), TG(16:1/14:0/17:1), and TG(15:0/14:0/18:2; *p* < 0.05), but significant negative correlations with TG(18:0/17:0/20:0), TG(16:0/17:0/24:0), TG(30:0/18:0/18:1), TG(30:1/16:0/18:0), TG(29:0/18:0/18:1), TG(18:0/18:0/18:3), and TG(19:0/18:1/18:2; *p* < 0.05). While g__*Rhodoblastus* was positively correlated with TG(18:1/18:1/22:5; *p* < 0.05), it was negatively correlated with TG(19:0/18:1/18:2; *p* < 0.05). The g__*Rhodoplanes* had positive correlations with TG(18:1/18:1/22:5), TG(16:0/17:0/ 18:1), TG(16:0/16:1/17:1), TG(15:0/14:0/16:1), TG(19:1/18:1/18:2), TG(16:0/14:0/14:0), TG(16:1/14:0/17:1), TG(15:0/14:0/18:2), and TG(20:0e/14:0/16:0; *p* < 0.05), but negative correlations with TG(18:0/17:0/20:0), TG(16:0/17:0/24:0), TG(30:0/18:0/18:1), TG (30:1/16:0/18:0), TG(29:0/18:0/18:1), TG(18:0/18:0/18:3), and TG(19:0/18:1/18:2; *p* < 0.05). For g__*Rhodopseudomonas*, it was positively correlated with TG(18:1/18:1/22:5), TG(16:0/17:0/18:1), TG(16:0/16:1/17:1), TG(15:0/14:0/16:1), TG(16:0/14:0/14:0), TG(16:1/14:0/17:1), and TG(15:0/14:0/18:2), TG(20:0e/14:0/16:0; *p* < 0.05) though negatively correlated with TG(18:0/17:0/20:0), TG(16:0/17:0/24:0), TG(30:0/18:0/18:1), TG(30:1/16:0/18:0), TG(29:0/18:0/18:1), TG(18:0/18:0/18:3), and TG(19:0/18:1/18:2; *p* < 0.05). Significant negative correlation with TG(15:0/16:1/18:3), TG(16:1/13:0/14:0), TG(16:0/17:0/18:1), and TG (18:0/18:1/20:3) were found for g__norank_f__*Barnesiellaceae* (*p* < 0.05). By contrast, g__norank_f__*Caldilineaceae* had positive correlations with TG(18:1/18:1/22:5), TG(16:0/17:0/18:1), DG(16:1/18:1), TG(16:0/16:1/17:1), TG(16:0/16:0/17:1), TG(15:0/14:0/16:1), TG(16:0/14:0/14:0), TG(16:1/14:0/17:1), and TG(15:0/14:0/18:2; *p* < 0.05), for which negative correlations with TG(18:0/17:0/20:0), TG(16:0/17:0/24:0), TG(30:0/18:0/18:1), TG(30:1/16:0/18:0), TG(29:0/18:0/18:1), TG(18:0/18:0/18:3), TG(19:0/18:1/18:2) were also present (*p* < 0.05). The g__norank_f__*Rhizobiales_Incertae_Sedis* had a positive correlation with TG(16:0/17:0/18:1; *p* < 0.05), as did g__norank_f__norank_o__*Chloroplast* with TG(18:1/18:1/ 22:5), TG(16:0/17:0/18:1), TG (18:0/18:1/20:3), and TG(16:0/16:1/ 17:1; *p* < 0.05) along with a negative correlation with TG(18:0/ 18:0/18:3) and TG(19:0/18:1/18:2; *p* < 0.05). For g__norank_f__norank_o__PeM15, it had positive correlations with TG(18:1/18:1/22:5), TG(16:0/17:0/18:1), TG (18:0/18:1/20:3), and TG(16:0/16:1/17:1; *p* < 0.05) but negative correlations with TG(16:0/17:0/24:0), TG(30:1/16:0/18:0), TG(18:0/18:0/18:3), and TG(19:0/18:1/18:2; *p* < 0.05). The g__norank_f__norank_o__RBG-13-54-9 was positively correlated with both TG(18:1/18:1/ 22:5) and TG(16:0/17:0/18:1; *p* < 0.05) and negatively correlated with TG(18:0/18:0/18:3; *p* < 0.05). Positive correlations with TG(18:1/18:1/22:5), TG(16:0/17:0/18:1), TG(16:0/16:1/17:1), TG(16:1/14:0/17:1), and TG(15:0/14:0/18:2; *p* < 0.05), as well as negative correlation with TG(18:0/17:0/20:0), TG(16:0/17:0/24:0), TG(30:1/16:0/18:0), TG(29:0/18:0/18:1), TG(18:0/18:0/18:3), and TG(19:0/18:1/18:2) were found for g__norank_f__norank_o__ *Saccharimonadales* (*p* < 0.05). With respect to g__unclassified_c__*Gammaproteobacteria*, it had positive and negative correlation, respectively, with TG(18:1/18:1/22:5) and TG(19:0/18:1/18:2; *p* < 0.05). Both g__unclassified_f__*Chloroflexaceae* and g__unclassified_f__*Chromatiaceae* had positive correlations with TG(18:1/18:1/22:5; *p < 0.05*), while the latter was negatively correlated with TG(19:0/18:1/18:2; *p* < 0.05). The g__unclassified_f__ *Rhodobacteraceae* was positively correlated with TG(18:1/18:1/22:5) and TG(16:0/17:0/18:1; *p* < 0.05) while negatively correlated with TG(18:0/18:0/18:3; *p* < 0.05). For g__ unclassified_f__*Xanthobacteraceae*, it had a positive correlation with the following: TG(18:1/18:1/22:5), DG(16:1/18:1), TG(16:0/16:1/17:1), TG(16:0/16:0/17:1), TG(15:0/14:0/16:1), TG(16:0/14:0/14:0), and TG(16:1/14:0/17:1), TG(15:0/14:0/18:2; *p* < 0.05). However, it had a negative one with these: TG(18:0/17:0/20:0), TG(16:0/17:0/24:0), TG(30:1/16:0/18:0), TG(29:0/18:0/18:1), and TG(18:0/18:0/18:3), and TG(19:0/18:1/18:2; *p* < 0.05). The g__unclassified_k__norank_d__Bacteria was negatively correlated with TG(15:0/16:1/18:3) and TG(16:1/13:0/14:0; *p* < 0.05), as was g__unclassified_o__ *Bacteroidales* with TG(15:0/16:1/18:3), TG(16:1/13:0/14:0), TG(16:0/ 17:0/18:1), and TG (18:0/18:1/20:3; *p* < 0.05). For g__unclassified_o__*Micrococcales*, it had positive correlations with TG(18:1/18:1/22:5), TG(16:0/17:0/18:1), TG(16:0/16:1/17:1), TG(15:0/14:0/16:1), TG(16:0/14:0/14:0), TG(16:1/14:0/17:1), and TG(15:0/14:0/18:2; *p* < 0.05) in addition to negative correlation with TG(18:0/17:0/20:0), TG(16:0/17:0/24:0), TG(30:1/16:0/18:0), TG(29:0/18:0/18:1), and TG(18:0/18:0/18:3; *p* < 0.05). The g__unclassified_o__*Rhizobiales* was positively correlated with TG(18:1/18:1/22:5), TG(16:0/17:0/18:1), TG(16:0/ 16:1/17:1), TG(16:0/14:0/14:0), TG(16:1/14:0/17:1), and TG(15:0/14:0/18:2; *p* < 0.05), but negatively correlated with TG(18:0/17:0/20:0), TG(16:0/17:0/24:0), TG(30:0/18:0/18:1), TG(30:1/16:0/18:0), TG(29:0/18:0/18:1), TG(18:0/18:0/18:3), and TG(19:0/18:1/18:2; *p* < 0.05). A positive correlation between g__*Chloronema* and TG(18:1/18:1/22:5) was found (*p* < 0.05). The g__*Christensenellaceae*_R-7_group had positive correlations with TG(18:1/18:1/22:5), TG(16:0/17:0/18:1), TG(16:0/16:1/17:1), and TG(16:1/14:0/17:1; *p* < 0.05) and negative ones with TG(16:0/17:0/24:0), TG(18:0/18:0/18:3), and TG(19:0/18:1/18:2; *p* < 0.05). Many correlations were significant for g__*Clostridium*_sensu_stricto_1, these being positive vis-à-vis DG(18:1/22:5), TG(15:0/20:5/22:1), TG(18:1/18:1/22:5), TG(16:0/17:0/18:1), TG (18:0/18:1/20:3), DG(16:1/18:1), DG(18:1/14:0), DG(18:3e/16:1), TG(19:1/16:0/18:0), TG(18:0/17:1/20:0), TG(16:0/16:1/17:1), DG(15:0/18:1), TG(16:0/16:0/17:1), TG(18:0/16:0/17:1), TG(15:0/14:0/16:1), TG(19:1/18:1/18:2), TG(16:1/14:0/17:1), and TG(15:0/14:0/18:2; *p* < 0.05); and negative vis-à-vis TG(18:0/ 17:0/20:0), TG(16:0/17:0/24:0), TG(30:0/18:0/18:1), TG(30:1/16:0/ 18:0), TG(29:0/18:0/18:1), TG(16:0/16:0/24:0), TG(26:0/16:0/17:0), TG(26:0/16:0/16:0), TG(28:0/16:0/16:0), TG(30:0/16:0/16:0), TG(28:0/16:0/17:0), TG(18:0/18:0/18:3), TG(19:0/18:1/18:2), WE (3:0/22:2), and WE(3:0/18:1; *p* < 0.05). By contrast, g__*Epulopiscium* only had a negative correlation with TG(16:1/13:0/14:0; *p* < 0.05). For g__Leptolyngbya_ANT.L52.2, there were positive correlations with TG(18:1/18:1/22:5), TG(16:0/17:0/18:1), DG(16:1/18:1), TG(16:0/16:1/17:1), TG(16:0/16:0/17:1), TG(15:0/14:0/16:1), TG(16:0/14:0/14:0), TG(16:1/14:0/17:1), and TG(15:0/14:0/18:2; *p* < 0.05) and negative correlations with TG(18:0/17:0/20:0), TG(16:0/17:0/24:0), TG(30:1/16:0/18:0), TG(29:0/18:0/18:1), TG (18:0/18:0/18:3), TG(19:0/18:1/18:2; *p* < 0.05). Finally, g__ *Macellibacteroides* was negatively correlated with TG(18:1/18:1/ 22:5), TG(16:0/17:0/18:1), DG(16:1/18:1), and DG(18:1/14:0; *p* < 0.05; Figure 9).

### Correlation Analysis of Lipidomics

Based on the correlations between microbiota and lipidomics data, TG(18:1/18:1/22:5) was chosen as the main reference lipid. We found that TG(20:0e/14:0/16:0), TG(16:0/17:0/22:0), WE(3:0/18:1), WE(3:0/18:2), TG(18:0/18:0/18:3), TG(19:0/18:1/ 18:2), TG(30:0/18:0/18:1), TG(30:1/16:0/18:0), TG(29:0/18:0/ 18:1), WE(3:0/24:2), TG(16:0/16:0/24:0), TG(18:0/17:0/20:0), TG(16:0/17:0/24:0), TG(26:0/16:0/17:0), TG(30:0/16:0/16:0), TG(28:0/16:0/17:0), WE(3:0/22:2), TG(26:0/16:0/16:0), TG(28:0/ 16:0/16:0), Hex1Cer(d18:1/18:0 + 2O), TG(16:0/6:0/11:3), and TG(16:0/6:0/9:0), TG(11:0/10:1/14:3) all had significant positive correlations with TG(18:1/18:1/22:5). Conversely, it was negatively correlated with TG(16:0/16:0/16:0), DG(18:1/22:5), DG (18:1/20:3), TG(15:0/20:5/22:1), TG(16:0/17:0/18:1), TG(18:0/ 18:1/20:3), TG(15:0/16:1/18:3), TG(16:1/13:0/14:0), TG(15:0/ 16:0/16:1), DG(15:0/18:1), DG(18:3e/16:1), DG(18:1/14:0), DG (16:1/18:1), TG(19:1/16:0/18:0), TG(16:0/16:1/17:1), TG(16:0/ 16:0/17:1), TG(18:0/16:0/17:1), TG(16:1/14:0/17:1), TG(15:0/ 14:0/18:2), TG(18:0/17:1/20:0), TG(19:1/18:1/18:2), TG(15:0/ 14:0/16:1), TG(16:0/14:0/16:0), TG(15:0/16:0/16:0), TG(15:0/ 16:0/18:3), and TG(16:0/14:0/14:0; Figure 10).

**Figure 10:**
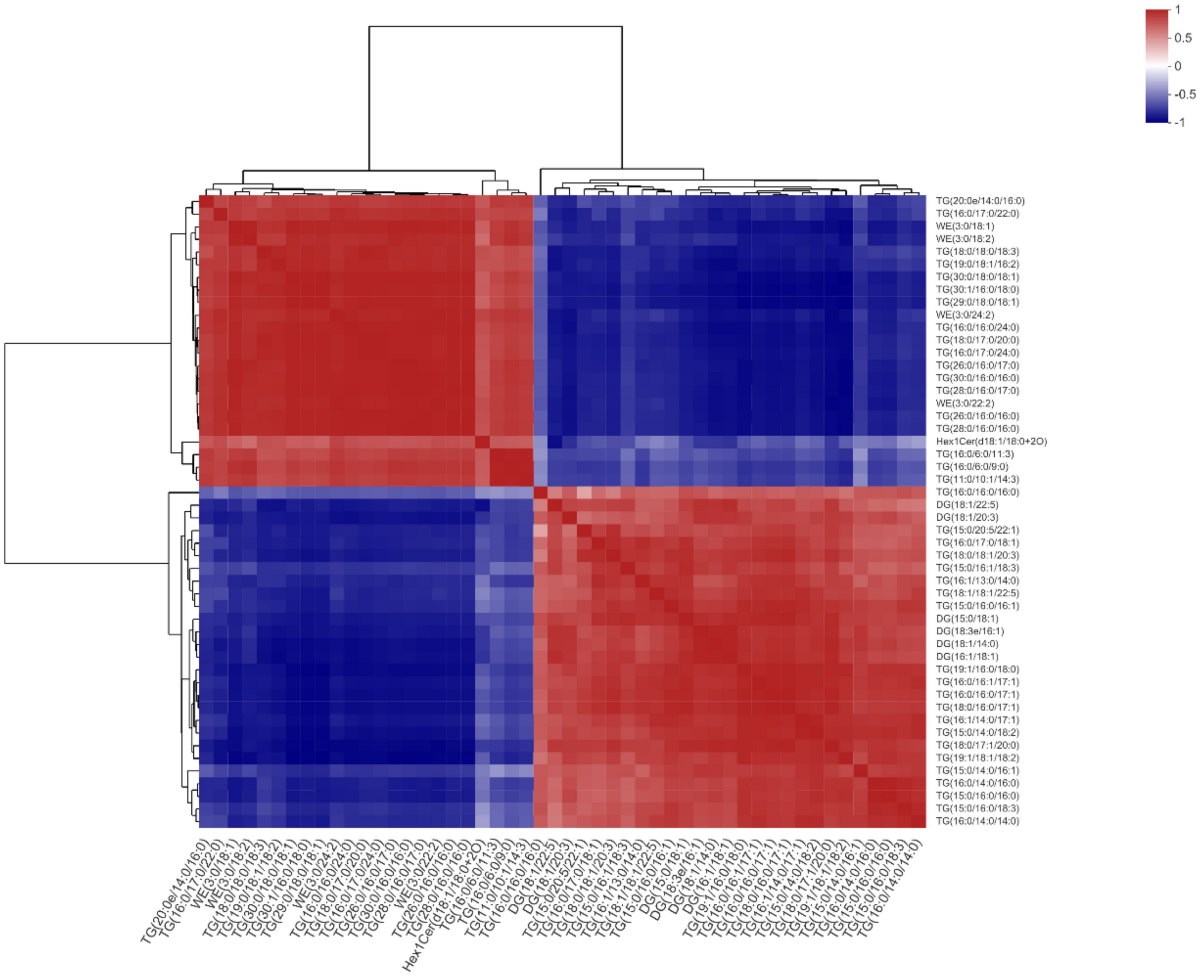
Effects of methionine restriction on the correlations between lipids after feeding for 8 weeks (*n* = 3).

### KEGG Pathway Analysis of Lipidomics

**Figure 11:**
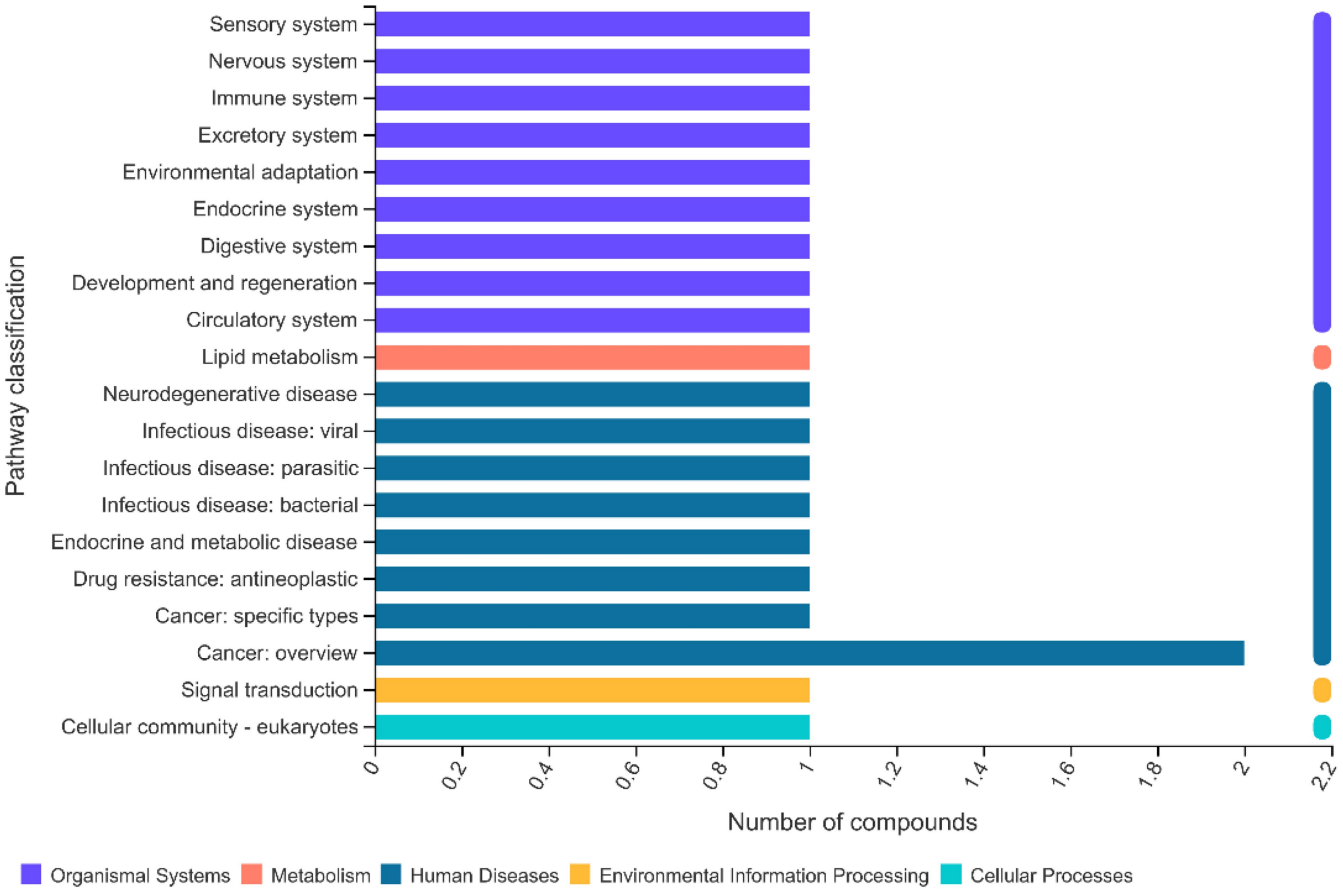
Effects of methionine restriction on the KEGG pathways of lipidomics data after feeding for 8 weeks (*n* = 3).

According to the KEGG results, the enriched pathways included lipid and glycan biosynthesis and metabolism, in addition to those associated with digestive, sensory, and immune systems (Figure 11).

## Discussion

Our previous studies indicated that a methionine-deficient diet not only impairs muscle fiber growth and the development and differentiation of *M. albus*, but it also weakens the eel’s overall growth performance (Hu et al., 2022b) and induces a lipid metabolism disorder resulting in a lowered lipid content of *M. albus,* mainly by impacting fatty acid metabolism (reduced fatty acids synthesis and increased fatty acid decomposition; Hu et al., 2021a). More recently, we showed that a diet deficient in methionine lessened the activity of major gastric-intestinal digestive enzymes (amylase, lipase, and trypsin), reduced the function of intestinal absorption and damaged the intestinal barrier, and decreased the ability of intestinal lipid and fatty acid transportation in *M. albus* (Hu et al., 2022b). To better understand the mechanisms by which methionine restriction affects gastrointestinal lipid digestion, transportation, and absorption in *M. albus*, we focused on the M0 (0 g/kg) and M8 (8 g/kg) treatment groups to characterize community succession of gut microbiota and how it regulates lipid mechanisms.

Here, compared with M0, the gut bacterial Sobs, Shannon, ACE, and Chao1 in the M8 group were significantly increased. This phenomenon indicated that restricting methionine intake lowered the diversity of gut microbiota and rendered the community more alike; hence, a suitable dietary level of methionine could promote the balance of gut microbiota of *M. albus*. Our results are similar to another study done on mice, where a suitable methionine supplementation improved the intestinal barrier functioning, inflammatory response, and lipid metabolism by regulating their gut microbiota (Yang et al., 2019a). In the present study, *Fusobacteria* was the dominant phylum and *Cetobacterium* the dominant genus in the gut of *M. albus* whether under M0 or M8. This finding is consistent with our previous study on *M. albus* (Peng et al., 2019a). Nonetheless, under M8, the relative abundances of *Fusobacteria* and of the genera *Cetobacterium* and *Lactococcus* decreased, while those of the *Firmicutes*, *Proteobacteria,* and *Chioroflexi* phyla, as well as *Clostridium* and *Streptococcus* genera, all increased. *Fusobacteria* are anaerobic gram-negative bacteria (Thurnheer et al., 2019) whose presence might promote fatty acids’ absorption (Semova et al., 2012), corroborating our previous study’s finding that dietary methionine improved intestinal fatty acid transportation and absorption (Hu et al., 2022a). *Cetobacterium* is an anaerobic gram-negative bacterium (Xie et al., 2021), being the dominant member of the gut microbiota of many freshwater fish species (Larsen et al., 2014; Peng et al., 2019b; Yang et al., 2019a, 2020), for which lipid metabolism is associated with the abundance of *Firmicutes* (Peng et al., 2019b; Hu et al., 2020a). This phenomenon suggests dietary differences in the level of methionine could influence the relative abundance of *Firmicutes*, thus regulating lipid metabolism of *M. albus* through gut microbiota dynamics (closely related to lipid metabolism).

Gut bacteria can form a complex ecological network that maintains the community’s dynamic equilibrium by each species interacting with others (Coyte et al., 2015). Here, the circos plot and ecology network revealed taxonomic composition and suite of interspecific interactions in the gut microbial community of *M. albus*. Evidently, the dominant phyla in M0 were *Firmicutes*, *Proteobacteria*, *Actinobacteria*, *Chloroflexi*, and *Cyanobacteria*; the dominant phyla in M8 were *Firmicutes*, *Proteobacteria*, *Actinobacteria*, *Chloroflexi*, and *Cyanobacteria*. This pattern of dominance was also similar between the networks of the two dietary methionine treatment groups. Our study indicates that methionine restriction induced gut microbiota dysbiosis, such that the predicted ecological network within microbial community was starkly affected by methionine-deficient diet, and interact among different gut microbial community (Herren and McMahon, 2018), eventually affecting the host’s metabolism. Modularity may be considered the degree to which a network could be divided into clearly delimited submodules; it also may be considered as functional units capable of performing an identifiable task (Peng et al., 2019a). Concerning our study, the many edges in the circos plots and networks indicated the interspecies interactions among different OTUs, with various OTUs from the same phylum found clustered within one module. Meanwhile, cooperative interactions were prevalent in the two networks, but competitive interactions predominated in the submodules of each bacterial network. By definition, the dominant gut microbiota constitutes a major component of the ecological networks in the two treatment groups; hence, the y occupied key positions in each network, whose higher average connectivity indicated a more complex ecological network (Herren and McMahon, 2018). Additionally, various OTUs from the same phylum were clustered within one module. Here, many OTUs among the dominant gut microbiota acted as connectors in the network, which indicated a pivotal ecological role fulfilled by the dominant microbiota (Zhou et al., 2011). In the ecological network, certain species act as structural and functional keystone species that maintain certain network properties (Olesen et al., 2007). The methionine-deficient diet not only changed the species that form the gut microbial community but also affected the nutrient metabolism performed by it (Ghanbari et al., 2015). In addition, the COG functional results predicted that gut microbiota are mainly involved in energy production and conversion, amino acid transport and metabolism, carbohydrate transport and metabolism, and lipid transport and metabolism of *M. albus* in both treatment groups. Compared with methionine-restricted group, nitrate reduction, fermentation, and chemoheterotrophy by gut bacteria in the methionine-supplemented group were lower, while their photoautotrophy, phototrophy, aerobic-chemoheterotrophy, and nitrate reduction functions were higher. This result indicated that supplementing dietary feed with a suitable level of methionine could reduce gastrointestinal tract inflammation, and improve the functioning of intestinal absorption (Kaewtapee et al., 2009).

Plotting the PCA and PLS-DA scores lets one visually display the classification of samples, such that the greater separation degree of the two groups, the more significant are their differences (Miehle et al., 2020). Here, the results indicated the main components of lipidomics differed between the methionine-supplemented and -restricted group. In addition, methionine supplementation decreased the content of fatty acids, especially of saturated fatty acids, such as TG(26:0/16:0/17:0), TG(28:0/16:0/16:0), TG(26:0/16:0/16:0), TG(30:0/16:0/16:0), and others. Fermentation in the methionine-supplemented group also decreased, implying that supplementation could improve the function of gastrointestinal digestion and reduce the remaining nutrients in the gut, thereby reducing the production of harmful substances (Gurry and Scapozza, 2020) and improving gut homeostasis, findings similar to another study on turbot (*Scophthalmus maximus* L.; Gao et al., 2019). We have already reported that a restricted methionine intake damages the intestinal barrier and reduces the lipid and fatty acid transportation in *M. albus* (Hu et al., 2022a). We inferred that a dietary change might promote the absorption ability for intestinal fatty acids, mainly for unsaturated fatty acids, thus leaving more partly saturated fatty acids in the gut content. Meanwhile, *Clostridium* genus had positive correlations with DG(18:1/22:5), TG(18:1/18:1/22:5), TG(16:0/17:0/18:1), TG(18:0/18:1/20:3), etc., and negative correlations with TG(18:0/17:0/20:0), TG(16:0/17:0/24:0), TG(16:0/16:0/24:0), etc. In addition, enriched KEGG pathways for metabolism mainly included those for lipid and glycan biosynthesis and metabolism, and those for digestive, sensory, and immune systems. Therefore, methionine restriction may affect lipid metabolism by mediating the succession of the gut microbiota community. Methionine restriction also inhibited the hepatic lipid deposition of *M. albus*, and chiefly downregulated hepatic fatty acid synthesis, especially for unsaturated fatty acids (C18:2*n* − 6, C22:6*n* − 3; Hu et al., 2021a). Furthermore, we found that g_*_Acetobacterium*, g__*Rhodoblastus* and g__*Clostridium*_sensu_stricto_1 each had a significant positive correlation with TG(18:1/18:1/22:5), the latter chosen from among the main lipids to investigate correlations with gut bacterial taxa and other lipids. Many kinds of saturated fatty acids, such as TG(16:0/17:0/22:0), TG(16:0/16:0/24:0), and TG(18:0/17:0/20:0), have significant positive correlations with TG(18:1/18:1/22:5), whereas most unsaturated fatty acids, such as [TG(15:0/20:5/22:1), TG(18:0/18:1/20:3), TG(15:0/16:1/18:3), and TG(19:1/18:1/18:2)] have significant negative correlations with TG(18:1/18:1/22:5). The mechanisms linking fatty acids metabolism and succession of gut microbiota community are highly complex and deserve further systematic study.

In conclusion, a methionine restriction diet disturbed the balance of gut microbiota by deteriorating the submodules of *M. albus*, which reduced the average connectivity and number of connectors while also decreasing competitive interactions within the ecological network; however, the composition of gut microbiota went unchanged. In stark contrast, methionine supplementation clearly improved the homeostasis of gut microbiota and lipid metabolism.

## Data Availability Statement

The original contributions presented in the study are publicly available. This data can be found at: https://github.com/mineraltsai/manuscript_FM.

## Ethics Statement

Our study was approved by Committee of Laboratory Animal Management and Animal Welfare of Hunan Agricultural University (Changsha, China) No. 094.

## Author Contributions

YaH was in charge of methodology, data curation, and writing—original draft. MC was in charge of data curation and software. WC was in charge of formal analysis and writing—review and editing. YiH was in charge of funding acquisition, writing—review and editing, and validation. All authors contributed to the article and approved the submitted version.

## Funding

This study was financially supported by National Natural Science Foundation of China (grant no. 32172986).

## Conflict of Interest

The authors declare that the research was conducted in the absence of any commercial or financial relationships that could be construed as a potential conflict of interest.

## Publisher’s Note

All claims expressed in this article are solely those of the authors and do not necessarily represent those of their affiliated organizations, or those of the publisher, the editors and the reviewers. Any product that may be evaluated in this article, or claim that may be made by its manufacturer, is not guaranteed or endorsed by the publisher.

## Footnotes

^1^ www.majorbio.com
